# The Future Dynamics of Long-Term Care Pressure in China’s Longevity Era: A Prediction Based on the Discrete-Time Markov Model

**DOI:** 10.3390/healthcare13233024

**Published:** 2025-11-23

**Authors:** Ran Feng, Yiting Tan, Jianyuan Huang

**Affiliations:** 1 School of Public Administration, Hohai University, Nanjing 211100, China; 220214140008@hhu.edu.cn; 2 Population Research Institute, Hohai University, Nanjing 211100, China; hhuhjy@hhu.edu.cn

**Keywords:** the longevity era, care pressure, demand prediction, person-years with disability

## Abstract

**Highlights:**

**What are the main findings?**

- The number of disabled older adults in China will peak at 160 million in 2070 and remain above 115 million by 2100, with the disability rate rising from 39.75% to 45.28%.
- Person-years with disability (PYD) will increase significantly among the oldest-old and women, highlighting sharp age and gender disparities in long-term care burdens.

**What are the implications of the main findings?**

- China’s long-term care system will face sustained and structural pressure, demanding urgent adaptation to demographic aging.
- Timely policy interventions are needed to mitigate future health risks and avoid a care crisis in the longevity era.

**Abstract:**

**Background**: In the era of longevity, many low- and middle-income countries (LMICs) still lack a comprehensive understanding of health deficits among older adults and the care burden associated with “unhealthy longevity”. This study aims to reveal future changes in care needs and pressure in China from 2030 to 2100. **Method**: This study develops a multistate demographic forecasting framework by integrating a Markov-based health state transition model with the conceptual logic of an age-shift algorithm. Transition probability matrices by age and gender are estimated using nationally representative microdata from the Chinese Longitudinal Healthy Longevity Survey (CLHLS). Baseline population data from the National Bureau of Statistics and WPP 2024 are then used to simulate the evolution of health status among older adults in China from 2030 to 2100. Finally, person-years with disability (PYD) are calculated to evaluate the projected magnitude, structure, and gender disparities of long-term care needs over time. **Results**: Between 2030 and 2100, the number of disabled older adults in China is projected to follow an inverted U-shaped trend—peaking at 160 million in 2070 and remaining above 115 million by 2100. The share of disabled individuals among older adults rises steadily, from 39.75% to 45.28%. Person-years with disability (PYD) show sustained growth, especially among the oldest-old and women. By 2100, adults aged 95 and older contribute over 20 million PYD—eight times the 2030 level. Gender disparities widen: in 2100, women aged 85–94 account for 53.94 million severe-disability PYD, exceeding men by 8.22 million. These trends reflect mounting structural pressures on China’s long-term care system, increasingly driven by age- and gender-specific disability burdens. **Conclusions**: If the current disability trend continues unchecked, health risks for older adults will grow over time. In the near future, China will face an extremely heavy care burden and pressure, which will severely impact its economic and social systems. Seizing this critical window for policy action and system improvement is crucial to reducing risks in the longevity era.

## 1. Introduction

Significant advances in medical science, public health programs, and social policies have noticeably prolonged the average lifespan of global humans [1]. According to the data from the United Nations’ World Population Prospects [2], life expectancy (LE) at birth reached 73.3 years in 2024, and the global average LE is projected to reach approximately 77.4 years by 2054. This indicates that an increasing number of individuals now have the potential to live into old age or even beyond. It is undoubtedly a great achievement. Never before in human history have so many people attained such long lifespans. However, an increase in LE does not necessarily correspond to a proportional extension of healthy life expectancy (HLE). A series of studies has shown that the prevalence of chronic (also referred to as lifelong) conditions, such as cardiovascular diseases, cancers, respiratory diseases, and rheumatoid arthritis, is rising [3,4], which has constituted the leading causes of mortality and disability worldwide [5]. In particular, many of these diseases are strongly associated with ageing. Some research has revealed that over two-thirds of adults aged 65 and above live with long-term chronic diseases [6,7]. As a result, health deficits among older adults and the resulting systemic challenges in long-term care (LTC) have emerged as critical research priorities in the era of longevity.

Currently, studies and predictions on older adults’ health and LTC pressure have achieved some progress, but there are still areas that have not yet been adequately understood. On the one hand, in the existing literature, most research on care pressure mainly focuses on trend judgments and subjective inferences [8,9], with a notable absence of systematic estimations related to the total volume of specific LTC demands. In other words, while it is generally recognized that the unhealthy and disabled lifespan is lengthening against the backdrop of a sustained extension in LE [10,11], there is still little quantitative evidence involving how much “care time” will be generated and how many “functional exposure years” will be accumulated in the process. Therefore, this study introduces an indicator of person-years with disability (PYD) based on the existing results of health status prediction as a direct response to this gap. More precisely, the core innovation of the study lies in its first attempt to explicitly quantify this structural concept of “care pressure”. By predicting the distribution of the population’s health status and the duration of these statuses, it captures the total time that disability status occupies within the life cycle of older adults, and figures the “absolute volume” of LTC pressure in specific numerical terms. This not only reveals the care costs behind longevity, but also transforms “care pressure” from an abstract proposition into an empirical index system characterized by measurability, comparability, and traceability.

On the other hand, there have been a number of projections and analyses in relation to the health status and care needs of older adults in the previous literature, but the majority of them are based on the contexts and experiences of high-income countries (HICs) [12,13]. Such as the United States, Germany, the United Kingdom, Italy [14,15,16]. Given that approximately 80% of older adults will reside in low- and middle-income countries (LMICs) over the next two to three decades [17], research on the assessment of future care needs for older adults and the care pressure in LMICs has been markedly underdeveloped. Especially, less scholarly attention has been devoted to China, a LMIC facing particularly severe challenges and pressures regarding care demands [18]. Data indicates that among LMICs, China has both a large older population (206.63 million) and a high aging level (15%), far exceeding that of India (103.69 million, 7%), Vietnam (9.14 million, 9%), and the Philippines (6.36 million, 5%); and its aging population will continue to grow at an accelerated pace in the coming decades [19]. For this, China is implementing comprehensive measures (including providing greater government investment, promoting market participation, and improving accessibility and supportiveness of care services) to strengthen its LTC systems. However, these efforts are hindered by multiple obstacles, one of which is an inadequate understanding of future care demands and associated systemic pressures [20]. Therefore, it is urgent to enhance relevant research rooted in the context of LMICs like China, so as to help them obtain additional information about their progress of aging population. And it can assist them in seizing the opportunity window for policy intervention and system design in time before more pressing problems or needs emerge [21]. Moreover, it is also valuable for facilitating comparisons and communication among regions with varying resource endowments, cultural norms, and institutional arrangements.

To summarize, this study proposes an index of person-years with disability (PYD) to more systematically measure the care pressure and longevity risk associated with the future disability status of the older population, based on the existing results of health status prediction. Unlike the Years Lived with Disability (YLD) indicator in the GBD framework, PYD does not incorporate severity weighting and is designed from a demographic and policy perspective, emphasizing population-wide care burdens over long time horizons. This distinction underscores the goal of PYD as a tool for projecting the volume and structure of long-term care demand, rather than evaluating disease-specific health losses. And then the study conducts a dynamic simulation for different degrees of PYD among various population groups in China from 2030 to 2100, by employing a Markov chain health-state transition model informed by the logic of an age-shift algorithm. Notably, in contrast to previous studies that have largely relied on regional samples or outdated data, this study adopts the age-specific population structure provided by the United Nations’ World Population Prospects 2024 (WPP 2024) [2] as its demographic baseline. This approach ensures internal methodological consistency and enhances the international comparability and credibility of the projections, thereby providing a basis for ascertaining the scale of China’s future LTC needs, care pressure, and policy orientation. Furthermore, it attempts to generate positive impacts on LMICs in the following aspects: fostering the building of an indicator framework to assess the magnitude of future care needs and care pressure; propelling the formulation of intervention measures to delay the onset of care needs and enhance healthcare and LTC systems [22,23].

## 2. Materials and Methods

### 2.1. Data Sources

This study draws on three primary data sources. The first is the Chinese Longitudinal Healthy Longevity Survey (CLHLS) conducted by Peking University. Among individuals aged 65 and above who participated in the five waves of the CLHLS conducted between 2000 and 2018, 10.2% participated in two waves, 1.5% in three waves, 0.2% in four waves, and only 0.1% in all five waves [24]. Given that population projections are typically more accurate for periods closer to the present [25]. Following studies such as Cui (2017) [26], which commonly use the two most recent waves to estimate health states transition probabilities, this study uses the 2014 and 2018 CLHLS follow-up data. The analytical sample includes men and women aged 65 and older. To ensure data quality, individuals with missing values on key variables were excluded, and cases lost to follow-up in 2018 were processed accordingly. The final sample comprises 5627 individuals, including 3421 surviving and 2206 deceased respondents. The second data source includes the Seventh National Population Census (hereafter referred to as “the 2020 Census”) and the 2024 revision of the World Population Prospects (WPP 2024) [2]. WPP 2024 provides data on China’s future demographic trajectories and complete life tables by age and sex, including projections of population size and life expectancy. The 2020 Census offers baseline data on the elderly population disaggregated by age and sex. We post-stratified the CLHLS analytical sample to the 65+ age–sex margins using the 2020 Census to align cohort sizes and the 2020 baseline health-state composition.

### 2.2. Definition of Health States

This study utilizes data from the 2014–2018 waves of the Chinese Longitudinal Healthy Longevity Survey (CLHLS) to construct transition matrices between health states and estimate the initial distribution of health conditions among older adults. The CLHLS covers 23 provinces across China and employs a multi-stage, stratified, random sampling strategy. It provides rich information on the physical health, family structure, cognitive status, and ability to perform activities of daily living of older adults. To ensure data quality, respondents with missing values on key variables were excluded, and those lost to follow-up in 2018 were addressed appropriately. The final analytical sample includes 5627 individuals, consisting of 3421 survivors and 2206 decedents.

This dataset was used for two primary purposes. First, we analyzed patterns of health state transitions among older adults using the 2014–2018 longitudinal subsample. After excluding cases with missing data and those with lost follow-ups, the valid sample size remained 5627. Health status was assessed using three core instruments: the Activities of Daily Living (ADL), the Instrumental Activities of Daily Living (IADL), and the Mini-Mental State Examination (MMSE) [27]. Higher numbers of independent ADL/IADL tasks indicate better physical functioning, while the MMSE score ranges from 0 to 30, with higher scores indicating better cognitive functioning. Following Shahid et al.’s classification [28], scores above 24 indicate good cognitive status, scores between 21 and 23 indicate moderate cognitive status, and scores below 21 indicate poor mental status.

Based on these indicators, health status was categorized into four groups: (1) healthy—no ADL or IADL limitations; (2) mildly impaired—1–2 ADL/IADL limitations; (3) functionally disabled—3 or more limitations or MMSE < 21; and (4) deceased. Among these states, all transitions are reversible except Death. The dynamic, continuous transitions across these three living states define the health transition process of older adults.

### 2.3. Construction of the Health Transition Model

(1) Markov Chain Assumption

The health status of older adults largely depends on their prior health conditions [29]. To capture this dynamic process, we adopt a discrete-time Markov chain framework to describe the transfer patterns of older adults among different health states, which assumes that the transition probability depends only on the current state and not on past states—implying the Markov property. Suppose that the likelihood of health state transition follows a time-homogeneous discrete Markov stochastic process on the health state space S and the age set T. The current health state is only related to the previous health state. Let the health transition process occur over a discrete set of time points $\{t_{m}{\}}_{m=1}^{T}\;$ within the finite state space $E=\left\{1,2,3,4\right\}$, representing Healthy, Mild Impairment, Functional Disability, and Death, respectively. Death is treated as an absorbing state. Under the Markov assumption, the conditional probability of transitioning to a future state depends only on the current state:

(1) $$P(X(t_{m+k})=j|X(t_{m})=i_{1},X(t_{m+1})=i_{2},\cdots ,X(t_{m+k-1})=i_{n})=P(X(t_{m+k})=j\;|X(t_{m+k-1})=i_{n})$$

Here, $t_{m}<t_{m+1}<\cdots <t_{m+k}\in T\;$, $i_{1},i_{2},\cdots ,i_{n}\in E$ and j∈E , the transition matrix is age and sex specific and estimated using CLHLS tracking data from 2014 to 2018. For each age group a, we define the time-varying transition probability matrix $p_{ij}^{\left(a\right)}(t)\;$ as follows:

(2) $$P^{\left(a\right)}(t)=\left[\begin{array}{cccc} p_{11}^{\left(a\right)}(t) & p_{12}^{\left(a\right)}(t) & p_{13}^{\left(a\right)}(t) & p_{14}^{\left(a\right)}(t)\\ p_{21}^{\left(a\right)}(t) & p_{22}^{\left(a\right)}(t) & p_{23}^{\left(a\right)}(t) & p_{24}^{\left(a\right)}(t)\\ p_{31}^{\left(a\right)}(t) & p_{32}^{\left(a\right)}(t) & p_{33}^{\left(a\right)}(t) & p_{34}^{\left(a\right)}(t)\\ 0 & 0 & 0 & 1 \end{array}\right]$$

where $p_{ij}^{\left(a\right)}(t)\;$ denotes the probability that individuals in age group a transition from state i to state j during interval t. Transitions from the absorbing state (Death) are fixed at 1, indicating that the system stays in the same state. Based on the 2014–2018 CLHLS longitudinal data, we estimate the initial transition probability matrix $P^{\left(a\right)}(4)\;$.

(2) Introducing Transition Strength to Construct the Health State Transition Probability Matrix

Since CLHLS data only allows calculation of the health state transition probability matrix  P(4)  over four years, it is difficult to adjust the prediction interval span freely. This paper draws on the concept of transition intensity introduced by Cui et al. (2017) [26], defined as the derivative of the transition probability matrix with respect to time t, denoted as:

(3) $$\frac{dP_{ij}}{dt}=\underset{\Delta t\to 0}{lim}\frac{P_{ij}(t+\Delta t)-P_{ij}(t)}{\Delta t}$$

where $P_{ij}\;$ denotes the probability of transitioning from state i to state j, and the transition intensity is defined as $\frac{dP_{ij}}{dt}=q_{ij}\;$. Expressing the transition intensity in matrix form $Q=[q_{ij}{]}_{n\times n}\;$, the Kolmogorov forward equation gives:

(4) $$P(t)=e^{\int_{t_{0}}^{t_{0}+t}Q(t)dt}$$

By calculating the transition intensity matrix $Q=[q_{ij}{]}_{5\times 5}$, and assuming constant transition intensities within one year, i.e., the transition rate is time-invariant, Equation (4) can be simplified as $P(t)=e^{Qt}$. Accordingly, the annual transition probability matrix is given by $P\left(t\right)=e^{Q}\;$.

In terms of age-grouping, this study adopts three primary considerations. First, individuals within the same decadal cohort (e.g., aged 60–69, 70–79, or 80 and above) are more likely to have experienced similar macroeconomic and social environments, exhibiting cohort effects in health transitions. Second, finer age groupings (e.g., 5-year intervals) may result in small subgroup sample sizes for specific health states, thereby reducing the precision and stability of transition probability estimates. Third, following the approach of Zhu [27], who grouped older adults in 10-year intervals (e.g., 64–74) to assess disability risk, this study also adopts 10-year age groups, assuming similar health distributional characteristics within cohorts. To ensure temporal consistency between age intervals and transition periods, we apply the matrix scaling method to derive the annual transition probability matrix from the 4-year transition matrix $P(10)=e^{10Q}=e^{\frac{10lnP(4)}{4}}\;$, thereby improving the accuracy of projections.

In terms of the stability and accuracy of health status projections, existing research suggests that in the absence of significant socioeconomic transformations or medical technology revolutions, the macro-environment exerts limited influence on health in the short to medium term [30]. Moreover, the health status of older adults within specific age groups tends to remain relatively stable over time [31]. Forecasts based on cohort and age-group changes are more accurate than those derived from cross-sectional trends [32]. Following the assumptions on health transition probabilities proposed by Cui (2017) [26] and others, this study establishes a predictive framework for the health status of older adults by age group and sex, based on data from the 2014–2018 waves of the CLHLS.

(3) Sensitivity analysis with scenario-specific time variation

To test the robustness of long-horizon projections, we allow post-2018 transition intensities to vary by calendar time under three scenarios that share the same identification anchors but differ in how the post-2018 drift is propagated. Let $P^{2011-2014}\left(3\right)\;$ (sourced from Zhu & Zhang [27]; same health-state taxonomy and age grouping as this study) be the three-year transition matrix around 2014, and $P^{2014-2018}\left(4\right)\;$ be the four-year matrix around 2018 estimated from the CLHLS panel. We map them to annualized generators using the principal matrix logarithm,

(5) $$Q^{2014}a,g=\frac{1}{3}\log\left(P^{2011-2014}a,g\left(3\right)\right)$$

(6) $$Q^{2018}a,g=\frac{1}{4}log\left(P^{2014-2018}a,g(4)\right)$$

With Death kept absorbing and diagonals set by row-sum zero, Transition probabilities for any span Δ are recovered by $P\left(\Delta \right)=\exp\left(Q\Delta \right)\;$.

We summarize the observed pre-2018 change by the per-year log-relative drift in each off-diagonal intensity,

(7) $$b_{ij}(a,g)=\frac{1}{4}log\left(\frac{max\{q^{2018}ij(a,g),\varepsilon \}}{max\{q^{2014}ij(a,g),\varepsilon \}}\right),\;\;\;\;i\neq j$$

where ε>0  secures numerical stability. For t≥2018 , scenario-specific off-diagonal intensities are parameterized as

(8) $$q^{S}ij(a,g,t)=q^{2018}ij(a,g)exp\left(\kappa^{S}b_{ij}(a,g)\phi (t)\right)$$

where $S\in \{\mathrm{Pessimistic},\mathrm{Neutral},\mathrm{Optimistic}\};\kappa^{S}\;$ controls how the historical drift is propagated, and ϕ(t)  governs its accumulation over calendar time. In pessimistic settings, we assume no technological progress or system improvements, hence $\kappa^{\mathrm{Pes}}=0\;$ (intensities remain at their 2018 level). The neutral scenario continues the observed trend with $\kappa^{N}=1\;$. The optimistic scenario retains the sign of the historical drift but modestly amplifies its magnitude with $\kappa^{\mathrm{Opt}}=1+\gamma \;$ for a small γ>0. After updating off-diagonals, diagonals are recalculated to satisfy $\sum_{j}q_{ij}^{S}\left(a,g,t\right)=0\;$; the Death row remains absorbing. This multiplicative formulation preserves non-negativity of off-diagonal intensities and conforms to the Kolmogorov forward link $P\left(\Delta \right)=\exp\left(Q\Delta \right)\;$.

Regarding the time accumulation function, with two historical anchors, it is not defensible to identify the slope and asymptote of logistic/Gompertz curves. We therefore adopt a transparent log-linear continuation ϕ(t)=t−2018  as baseline, and report a tempered alternative $\phi_{T}\left(t\right)=\min\left\{\frac{t-2018}{T_{max}},1\right\}\;$ in robustness checks, which allows the drift to accumulate linearly up to horizon $T_{max}\;$ and then plateau. This design maintains near-term dynamics data consistency while preventing unbounded long-run extrapolation when only two pre-2018 points are available. For any node year t and span Δ (in years), we compute $P^{S}a,g\left(t,\Delta \right)=\exp\left(Q^{S}a,g\left(t\right)\Delta \right)\;$, evaluate $P^{S}\left(4\right)\;$ at decade nodes (2020, 2030, …, 2100), and, in the population module, convert to $P^{S}\left(10\right)\;$ to match the decadal projection step without altering the downstream engine. The above is fully coherent with our Markov framework and the intensity-to-probability mapping described in Section 2.3(1).

(4) Population Projection Model by Health Status

Let the health states for older adults be denoted as $S=\left\{H,I,D,Death\right\}\;$, where H refers to healthy, I to mildly disabled, D to severely disabled, and Death to the absorbing state. Define $N_{s}^{\left(a,g\right)}\left(t\right)$ as the population in health state s∈S  at time t, for the age group $a\in \left\{65,75,85,95^{+}\right\}\;$ and gender $g\in \left\{M,F\right\}\;$. The initial distribution of health states is calibrated using CLHLS data:

(9) $$\pi^{\left(a,g\right)}s(t_{0})=\left(\frac{N^{\left(a,g\right)}H}{N^{\left(a,g\right)}},\;\frac{N^{\left(a,g\right)}I}{N^{\left(a,g\right)}},\;\frac{N^{\left(a,g\right)}D}{N^{\left(a,g\right)}}\right)$$

where $N^{\left(a,g\right)}=N^{\left(a,g\right)}H+N^{\left(a,g\right)}I+N_{D}^{\left(a,g\right)}\;$ represents the initial living population base. For each scenario $\sigma \in \left\{\mathrm{Pes},\mathrm{Neu},\mathrm{Opt}\right\}\;$, let $P_{\sigma }^{\left(a,g\right)}\left(\Delta \right)\;$ denote the scenario-specific transition probability matrix over span Δ years for age group a and gender g. In particular, the decadal (Δ=10) transition matrix takes the form:

(10) $$P^{\left(a,g\right)}\sigma (10)=\left[\begin{array}{cccc} p^{\left(a,g\right)}HH,\sigma & p^{\left(a,g\right)}HI,\sigma & p^{\left(a,g\right)}HD,\sigma & p^{\left(a,g\right)}H,Death,\sigma \\ p^{\left(a,g\right)}IH,\sigma & p^{\left(a,g\right)}II,\sigma & p^{\left(a,g\right)}ID,\sigma & p^{\left(a,g\right)}I,Death,\sigma \\ p^{\left(a,g\right)}DH,\sigma & p^{\left(a,g\right)}DI,\sigma & p^{\left(a,g\right)}DD,\sigma & p^{\left(a,g\right)}D,Death,\sigma \\ 0 & 0 & 0 & 1 \end{array}\right]$$

where the last row fixes Death as absorbing. The construction of $P^{\left(a,g\right)}\sigma \left(\Delta \right)\;$ from time-varying intensities is given in Section 2.3(2) and is not repeated here.

Using the 2018 CLHLS dataset and the scenario-specific annual transition matrix $P^{\left(a,g\right)}\sigma \left(1\right)$, we compute the proportion of older adults in each health state by age group and sex in 2020, denoted by $\pi^{\left(a,g\right)}s,\sigma \left(2020\right)\;$. Combining this with WPP 2024 population counts yields the baseline state-specific population vector for each sex–age group in 2020 under scenario σ:

(11) $$N^{\left(a,g\right)}s,\sigma \left(2020\right)=N^{\left(a,g\right)}\left(2020\right)\cdot \pi^{\left(a,g\right)}s,\sigma \left(2020\right),\;\;\;\;s\in \left\{H,I,D\right\}$$

For the 65–74 age group in projection years t≥2030 , we obtain the projected total population by sex and age from WPP 2024 and assign initial health states according to the 2020 composition under the same scenario; that is, we use $\pi_{s,\sigma }^{\left(65,g\right)}\left(2020\right)\;$ as the initial composition for newly entering cohorts aged 65–74 in each period.

For a≥75 , we adopt the Markov update using the scenario-specific decadal matrix. Taking the 75–84 group as an example, its state vector evolves from the surviving population of the preceding decade’s 65–74 group through

(12) $$\left(N^{\left(75,g\right)}H,\sigma \left(t\right),N^{\left(75,g\right)}I,\sigma \left(t\right),N_{D,\sigma }^{\left(75,g\right)}\left(t\right)\right)\left(N^{\left(65,g\right)}H,\sigma \left(t-10\right),N^{\left(65,g\right)}I,\sigma \left(t-10\right),N^{\left(65,g\right)}D,\sigma \left(t-10\right)\right)\cdot P^{\left(65,g\right)}\sigma \left(10\right)$$

For the 95+ age group, the projected population is the sum of two components: survivors from the 85–94 age group in the previous decade and those who were already in the 95+ age group at the beginning of the decade. Under scenario σ, this yields

(13) $$\left(N^{\left(95,g\right)}H,\sigma (t),N^{\left(95,g\right)}I,\sigma (t),N_{D,\sigma }^{\left(95,g\right)}(t)\right)\left(N^{\left(85,g\right)}H,\sigma (t-10),N^{\left(85,g\right)}I,\sigma (t\;-10),N^{\left(85,g\right)}D,\sigma (t-10)\right)\cdot P^{\left(85,g\right)}\sigma (10)\;+\left(\left(N^{\left(95,g\right)}H,\sigma (t-10),N^{\left(95,g\right)}I,\sigma (t-10),N^{\left(95,g\right)}D,\sigma (t-10\right)\right)\;\cdot P^{\left(95,g\right)}\sigma (10)$$

(5) Estimating Care Time Demand for Elderly People in Different Disability States

The estimation of care time demand for different disability states is based on the duration spent in each health state. This study adopts the Sullivan method and introduces the concept of person-years lived = $\sum \left(number\;of\;people\times years\;lived\right)$, to examine how long older adults remain in different disability states. Using sex- and age-specific life table data from WPP 2024, the complete life expectancy and average remaining life expectancy for each sex-age group are obtained. This allows the calculation of the number of survivors $l_{x}^{a}\;$ at age *x*, and the corresponding person-years lived $L_{x,i}^{a}\;$. According to the Sullivan method, the average duration spent in each disability state by people aged x is calculated as:

(14) $$e_{x,i}^{\alpha }=\frac{\sum_{65}^{\omega }L_{x,i}^{\alpha }}{l_{x}^{\alpha }},\;\;\left(\omega \;\mathrm{is}\;\mathrm{the}\;\mathrm{maximum}\;\mathrm{age}\right)$$

where α, i and x denote sex, disability state and age group, respectively, and $l_{x}^{a}$ is the number of survivors.

(6) Estimating person-years with disability (PYD) Among old adults

This study proposes the indicator of person-years with disability (PYD), which quantifies the cumulative exposure time of elderly individuals in different disability states.

The PYD is mathematically defined as:

(15) $$PYD_{a,t}^{\left(s\right)}=N_{a,t}^{\left(s\right)}\times D_{a,t}^{\left(s\right)}$$

where a denotes the age group, t the year, and s the disability state. $N_{a,t}^{\left(s\right)}\;$ represents the number of individuals in age group a and state s at time t, while $D_{a,t}^{\left(s\right)}\;$ reflects the expected duration of state s, indicating the average time an individual remains in that state. The logic behind this indicator is both straightforward and intuitive: multiplying the number of individuals in a given health state by the expected duration of that state yields a population-level estimate of care time demand. Methodologically, this approach builds upon the Sullivan method. The Sullivan method estimates state-specific life expectancy by combining life table survival probabilities with age-specific prevalence of health states. It provides a practical and policy-relevant tool for calculating the years lived with different health conditions, without relying solely on mortality-based data. Notably, while the commonly used YLD (Years Lived with Disability) metric in the Global Burden of Disease (GBD) framework is derived from disease prevalence and severity weights, PYD is not adjusted by disability weights and is therefore not used for health valuation, but rather for projecting long-term care demands. The PYD framework proposed in this study shares the core logic of the Sullivan method but offers a simplified and interpretable extension. It enables dynamic estimation of health state exposure under population aging, offering theoretical and empirical support for long-term care system planning and healthy longevity risk assessments.

## 3. Results

### 3.1. Transition Probabilities Across Health States

**Figure 1:**
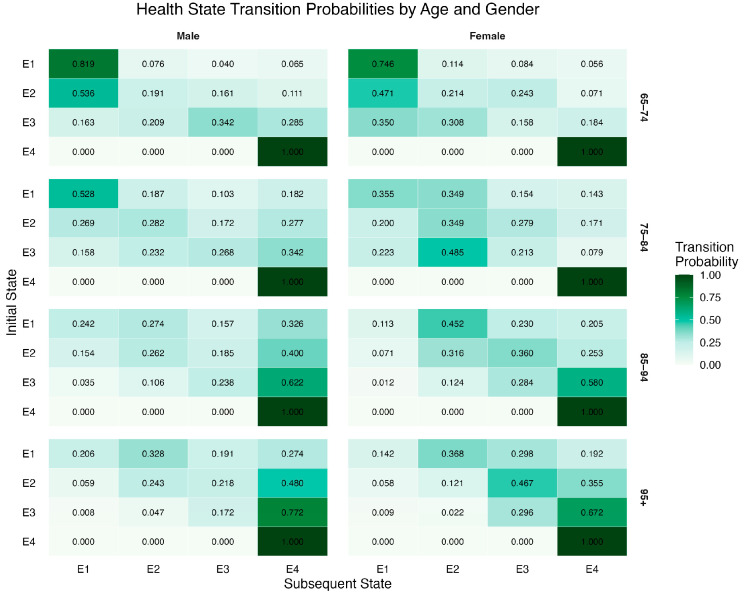
Age- and Sex-Specific Transition Probability Matrices of Health Status (Including Death) Among Older Adults. Source: Calculated using health status data of older adults from the 2014–2018 waves of the Chinese Longitudinal Healthy Longevity Survey (CLHLS). Note: E1, E2, E3, and E4 represent healthy, mildly disabled, severely disabled, and deceased states, respectively.

This study estimates the transition probability matrices of health states among older individuals across different subpopulations (see Figure 1), where each element represents the probability of transitioning from one health state to another within 4 years. The results indicate a transparent age gradient in health state transitions, with pronounced gender differences. Overall, as age increases, the probabilities of transitioning into disability or Death rise significantly, while the likelihood of maintaining good health or recovering to a better state gradually declines [33,34].

First, regarding transitions into mild disability, the probability of entering this state increases with age and is consistently higher for females across all age groups. The gender gap peaks in the 75–84 age group, with females showing a 0.178 higher probability than males, suggesting greater vulnerability among older women to functional decline. For individuals already in the mildly disabled state, the likelihood of remaining in this state shows an upward trend with age among males (from 0.1909 in the 65–74 group to 0.2434 in the 95+ group), but a declining trend among females (from 0.2143 to 0.1206). This pattern suggests that older women are more likely to deteriorate after entering mild disability, indicating a higher risk of long-term care dependency and reflecting the well-documented “longer life but poorer health” phenomenon among females [35,36]. Recovery from mild disability to full health is rare across all age groups and declines sharply with age, with a probability of only 0.036 in the 95+ group. This finding highlights the minimal potential for functional recovery among the oldest-old.

Second, regarding transitions into severe disability, the probability of entering this state rises rapidly with increasing age and the worsening of the initial health condition, with a particularly steep increase observed among women. For instance, the probability of transitioning from mild to severe disability rises from 0.1613 to 0.2176 among men aged 65–74 and 95+, respectively. In contrast, the corresponding probability for women increases from 0.2429 to 0.4667, which is nearly twice that of men. By age 95, women’s probabilities of entering severe disability from healthy and mildly disabled states exceed those of men by 0.107 and 0.249, respectively, highlighting the accumulated exposure of the oldest-old women to high-risk conditions of profound disability. The probability of remaining in a state of severe disability also increases markedly with age and tends to stabilize in advanced age. For men, it rises from 0.3424 to 0.7724 between the 65–74 and 95+ groups, and for women, from 0.3083 to 0.6812. These findings indicate that once individuals enter a severely disabled state, they are likely to remain in it for the rest of their lives, with limited potential for intervention or recovery [37].

Finally, the probability of transitioning into Death increases significantly among those aged 85 and above, exhibiting a strong age-related pattern. Men face consistently higher mortality risks under disabled conditions than women, which may be attributed to a higher incidence of acute health events and the lack of spousal support [38,39]. In contrast, although women exhibit relatively lower mortality rates under severe disability, they are more likely to remain in highly disabled states for extended periods, resulting in a greater demand for long-term care. This differentiated pattern, which is characterized by higher mortality in men and longer durations of disability in women, underscores the necessity of policy attention to severely disabled older women in the design of care systems.

**Figure 2:**
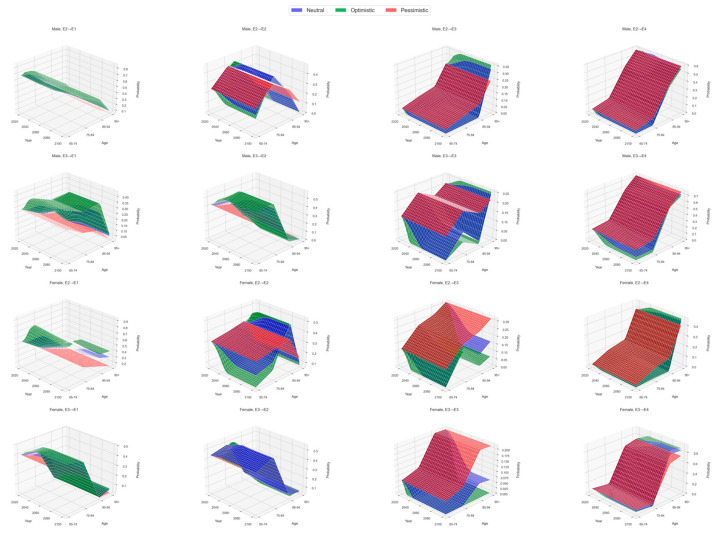
Scenario-based projections of disability-state transition probabilities by age and sex, 2020–2100. Data sources: Chinese Longitudinal Healthy Longevity Survey (CLHLS) 2011, 2014, and 2018 tracking waves; authors’ calculations. Scenario definitions follow Section 2.3(3).

Beyond the static estimates based on the 2014–2018 matrix, we further project with time-varying transition matrices under three scenarios. The pessimistic scenario assumes that future years will continue to follow the 2014–2018 transition pattern without any medical or system improvements. The neutral scenario extends the historical trend, while the optimistic scenario slightly strengthens that trend in the same direction. Figure 2 focuses on transitions that start from disability states only—namely, movements from mild disability to recovery, to severe disability, or to Death, and movements from severe disability to improvement or to Death—by age group and sex from 2020 to 2100. Across panels, the optimistic case shows noticeably higher chances of recovery from mild disability and lower risks of worsening or Death from both mild and severe disability; the pessimistic case exhibits the opposite pattern, while the neutral case lies in between.

### 3.2. Projected Population Size of Older Adults with Different Levels of Disability

As shown in Table 1, between 2030 and 2100, the size of the disabled older adult population in China is projected to follow a phased trajectory characterized by rapid growth in the early period and a gradual decline thereafter, with mounting pressure on care systems in both absolute and structural terms. In absolute numbers, the population of older adults with disabilities is projected to rise from 82.38 million in 2030 to a peak of 152.81 million by 2050, representing an average annual growth rate of approximately 3.4%. This phase marks a period of rapid expansion in care needs, placing the most significant strain on the care system. Although a decline follows, the overall level remains high, with 115.11 million projected by 2100, which is substantially above the 2030 baseline. This highlights the long-term and lagging nature of care burdens.

The relative proportion also shows an upward trend. The proportion of disabled individuals among the older adult population is expected to rise from 39.75% in 2030 to 45.28% in 2100, indicating that nearly one in every two older adults will experience some degree of disability. This structural transformation implies that even if the total older population decreases to some extent, the share of those requiring care will continue to grow, resulting in heavier care burdens per capita. Notably, between 2050 and 2080, the proportion remains consistently above 41%, peaking at 44.64% in 2080, signaling sustained and high-density demand for care services. In sum, both the scale and structural proportion of the disabled elderly population suggest that China will face long-term and structural challenges in its care system.

**Figure 3:**
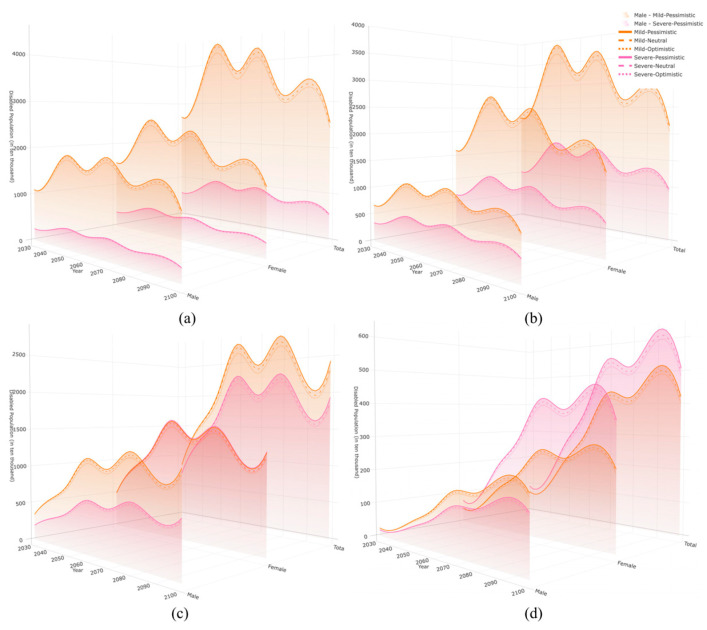
Scenario-based projections of the disabled older population in China, by age group, sex, and disability severity, 2030–2100 (millions). Note: Panels (**a**–**d**) correspond to age groups 65–74, 75–84, 85–94, and 95+, respectively. Within each panel, the three slices labeled “Total,” “Male,” and “Female” show the overall and sex-specific populations. Colored surfaces depict pessimistic, neutral, and optimistic scenarios; color families distinguish mild and severe disability (see legend). Shaded envelopes indicate the magnitudes of the scenarios, and overlaid lines trace the corresponding time trajectories.

Figure 3 presents the projected trends for disabled older adults, categorized by disability type (mild and severe), and by age group (panels a to d), under the pessimistic scenario. The total number increases with both age and time, reaches a peak around the middle of the century, and then declines slightly. Although the 65–74 group is the youngest segment, its large base is expected to produce nearly 30.00 million disabled individuals by 2030, with a peak around 2050, followed by a modest decline. The 75–84 group grows quickly, and the number of severely disabled individuals reaches 17.44 million by 2050 and remains high thereafter. The 85–94 group becomes the focal point of the disability burden, with severely disabled individuals peaking at 22.87 million by 2060. The 95+ group starts from 911,800 in 2030 and climbs rapidly to 5.06 million by 2100, indicating a sharp accumulation of intensive care needs among the oldest-old. These patterns show that the oldest-old are increasingly central to the long-term care system and that the expanding size of the severely disabled population is a key dimension of longevity risk.

The figure also reveals pronounced gender differences: across all age groups and disability states, the number of disabled women consistently exceeds that of men, with the gap widening as age increases. For example, under the pessimistic scenario, in 2030, the number of disabled women aged 65–74 is 18.83 million compared with 13.46 million men. Among those aged 95+, disabled women number 1.19 million, nearly three times the male count. The disparity persists to 2100, with 5.80 million disabled women versus 3.47 million men. The gap is most evident in severe disability: in 2030, severely disabled women number 724,900, which is 3.88 times the male figure; by 2100, the ratio remains 2.34. This structural pattern reflects the combination of higher female life expectancy and longer time lived with disability, often described as female longevity with higher health vulnerability [40]. Women account for a larger share of the disabled population, enter the growth phase earlier, remain at elevated levels for longer, and are disproportionately concentrated in the 85+ range [41]. These results provide a structural basis for assessing future caregiving pressures and suggest prioritizing support for severely disabled older women in service planning.

### 3.3. Projected Duration of Disability States in Older Adults

The duration of disability serves as a key indicator of the pace of health decline in older adults. It constitutes a core determinant of the intensity and continuity of long-term care needs. With rising life expectancy, the period of disability has not been compressed; instead, it demonstrates a trend toward the expansion of morbidity [42]. Projections (see Appendix A Table A1) show that from 2030 to 2100, the average duration of both mild and severe disability steadily increases, particularly among the oldest-old population. For instance, among those aged 75–84, the average duration of mild disability rises from 4.48 to 5.93 years, and severe disability from 1.44 to 1.90 years.

Among adults aged 85 and above, the duration of healthy years gradually decreases, while the period of disability lengthens. In the 95+ age group, the duration of severe disability rises from 2.18 to 2.73 years, accounting for a significantly greater proportion of remaining life expectancy. Distinct patterns are observed across age groups. For example, older adults aged 65–74 have the most extended healthy lifespan, increasing from 11.93 to 15.24 years; yet, their duration of severe disability also increases from 1.19 to 1.53 years, indicating that extended longevity does not necessarily translate into an extended healthy lifespan.

Gender differences persist throughout the projection period, with women consistently experiencing longer durations of disability than men, and the gap widening over time. In 2030, women aged 65–74 are projected to experience 5.79 years of mild disability and 1.44 years of severe disability—both longer than men at 4.41 and 1.00 years, respectively. By 2100, these figures increase to 7.59 and 2.01 years, with the gender gap expanding to 2.16 and 0.48 years. Among the 75–84 and 85–94 age groups, women’s severe disability periods exceed those of men by nearly one year, indicating earlier onset and longer persistence of disability. These findings reflect a gendered risk structure commonly described as the “female longevity–morbidity paradox” [43,44].

### 3.4. Projected Scale of Disability Person-Years Across Different Disability States

**Figure 4:**
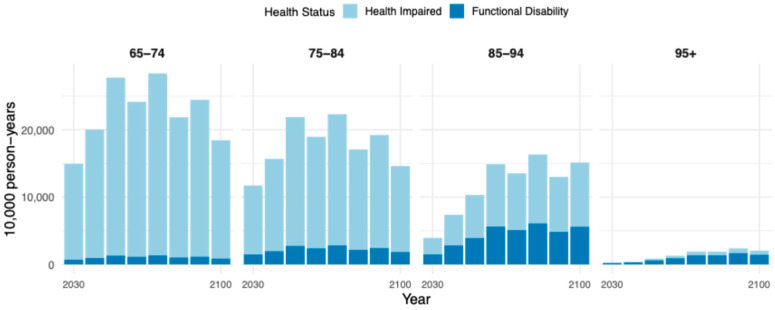
Person-Years of Population by Disability Status.

The indicator of person-years with disability (PYD) provides an integrated measure of the magnitude and duration of the disability burden. We compute PYD using the population projected under the pessimistic scenario to give a conservative upper bound and a cautionary benchmark, thereby reducing the risk of underestimating long-term care needs and supporting stress-test-style planning. Figure 4 shows that over the projection period, China’s older adult PYD is expected to grow before stabilizing, peaking around 2050, and then slightly declining, reflecting the combined effects of demographic shifts and transitions in health status. By age group, older adults aged 65–74 contribute the largest share of PYD in the early projection years, peaking at 277.48 million person-years in 2050, and then decreasing to 184.50 million in 2100. The 75–84 age group shows steady growth, while the 85–94 group exhibits a pronounced increase, reaching 151.35 million person-years by 2100. The 95+ age group shows the fastest growth, from 2.76 million person-years in 2030 to 20.39 million in 2100, indicating that the weight of the oldest-old in long-term care resources will continue to rise. By disability type, mild disability (care needs due to impaired health) remains the most prevalent condition. Among the 65–74 age group, the number of person-years with mild disability rises from 142.56 million in 2030 to 264.44 million in 2050, then declines to 175.81 million by 2100. Severe disability, in contrast, remains at a relatively low level for this group. However, among the 85+ population, severe disability person-years increase rapidly. For example, in the 85–94 group, the number rises from 14.95 million to 56.08 million, and in the 95+ group, from 1.99 million to 14.36 million between 2030 and 2100. These trends suggest that with advancing age, the risk and cumulative burden of severe disability increase, making it a significant source of care pressure for the oldest-old. This underscores the urgent need to prioritize service provision for high-age, high-burden groups, particularly those experiencing severe disability, and to develop a tiered and stratified care system capable of meeting the escalating demand associated with China’s rapidly aging population.

**Figure 5:**
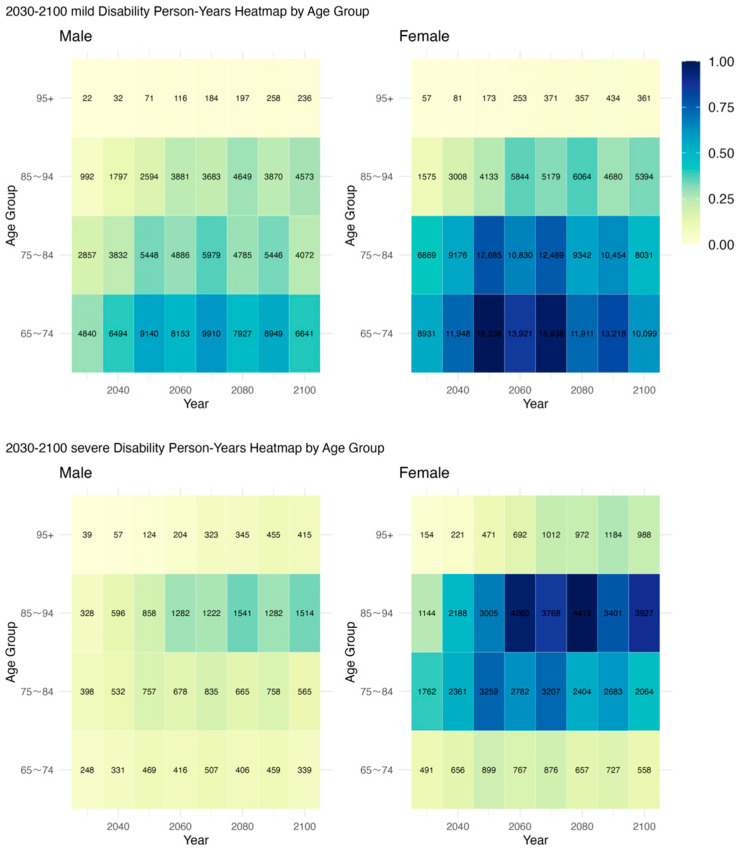
Disability person-years by severity and gender, 2030–2100. Note: Cell values denote 10,000 person-years. The color bar represents a 0–1 rescaled intensity for each severity level to facilitate visual comparison; darker shades indicate higher values. conditions.

The prediction results shown in Figure 5 indicate that pronounced gender disparities persist throughout the projection period in terms of disability person-years. Women consistently exhibit higher total person-years than men, and the gender gap widens with advancing age. By 2100, women aged 75–84 are projected to experience 80.31 million person-years of mild disability, nearly double that of men (40.72 million). In the 85–94 age group, women will face 53.94 million person-years of severe disability, compared to 45.73 million for men. This disparity is partly attributable to women’s longer life expectancy, but may also reflect their higher cumulative exposure to disability risk at advanced ages. Notably, in the 65–74 age group, the gender gap in mild disability burden is relatively modest: 89.31 million for women versus 66.41 million for men. However, this gap expands markedly in older age groups, indicating that the feminization of the elderly population will translate into greater gender-specific pressures on long-term care systems. Further insights from heatmap analyses reveal that male disability burden is more concentrated within the 75–84 age range, whereas female disability burden is denser and more prolonged in the 85+ age group. After 2060 in particular, women exhibit large-scale, high-density accumulations in both mild and severe disability person-years. For instance, in 2060, women aged 85–94 account for 58.44 million person-years of mild disability and 42.60 million of severe disability, both significantly exceeding their male counterparts. These trends underscore the necessity of enhanced care provisions for the oldest-old female population. In sum, the projected trajectory of disability among China’s older adults is characterized by sustained high volumes, a marked concentration in advanced ages, and a disproportionate burden borne by women. Facing the structural challenges of long-term care, it is essential to strengthen support systems for severely disabled, older women, direct care resources toward high-risk population segments, and develop a long-term care system with greater flexibility and structural responsiveness.

## 4. Discussion

This study predicts the development trends of the older population with health impairments and functional disabilities, the duration of these impairments and disabilities, as well as the volume of LTC demands in China from 2030 to 2100, using a Markov chain health-state transition model informed by the logic of an age-shift algorithm. The potential longevity risks and associated care burdens that China may face in the future are presented through more accurate, direct, and tangible data. It can provide new evidence for China to reevaluate and reform deficiencies in the current healthcare and LTC system, and may thereby offer valuable insights for LMICs globally to tailor health- and care-related plans [45,46].

### 4.1. The Continuous Increase in Older Population with Impairment and Dysfunction

This study showed that the number of older adults in China with health impairment and dysfunction is projected to increase from 82.38 million in 2030 to 115.11 million by 2100, with the corresponding proportion fluctuating from 39.75% to 45.28% over the same period. These results differ from those of previous studies [47], yet the observed trends are consistent. Health impairment and functional disability have been intractable issues confronting older adults in China and will persist as such in the future [48], which will profoundly constrain the improvement of life quality and well-being among older adults in the era of longevity.

China attained demographic aging in only approximately 27 years—a process that took HICs over 45 years, placing the country under increasing pressure from rapid population aging and a heavy burden of chronic diseases [49]. Against this backdrop, China has actively advanced the “Healthy China initiatives”, enhanced the accessibility and convenience of primary healthcare and LTC services, promoted the integration of medical care and LTC, and implemented the construction of age-friendly environments, demonstrating sustained coordinated efforts to comprehensively safeguard the health of older adults and advance healthy aging. It is encouraging that residents’ health literacy has shown a measurable improvement. The health literacy level among Chinese residents reached 31.87% in 2024, representing a 23.07 percentage points increase from the monitoring baseline in 2012 (8.80%) [50]. It is concerning that the growth in health literacy has yielded limited health benefits for older adults. In China, more than 190 million older adults were affected by chronic diseases [51]. Obvious advantages in HLE are not gained by this group, and the expansion of disability alongside the coexistence of multiple diseases was a prevalent phenomenon among them [52,53,54]. One study has indicated that the prevalence of multimorbidity among middle-aged and older adults in China reaches 56.73%, a figure substantially higher than the 37.3% reported for adults aged 50 and above across 16 European countries [55]. This is because the improvement of health literacy and the implementation of disease prevention and health promotion measures possess a “time-sensitive intervention windows” [56]. During this period, leveraging neuroplasticity and the formation of behavioral habits can yield substantial benefits for enhancing people’s health conditions. Conversely, beyond this “time-sensitive intervention windows”, the effectiveness of intervention measures decreases significantly. According to neuroimaging studies and the theory of persistence, middle-aged adults maintain a remarkable ability to form habits; older individuals exhibit a stronger dependence on their deeply rooted, long-standing living patterns and behavioral patterns [57]. Therefore, this study argues that the core strategy in China must undergo a strategic transformation: shifting from focusing on supporting the older population to continuously tracking individuals’ life courses and attending to how people grow, develop and age. It is imperative to make forward-looking investments in health promotion, disease prevention, and early intervention across the full life course. This represents a fundamental pathway to effectively reshape the aging trajectory, relieve future systemic pressures, and achieve the goal of “healthy longevity”. This perspective has been widely supported by researchers [58,59,60]. Otherwise, transformations in the disability model are likely to remain limited, and the projections from this study may well materialize in the future.

Meanwhile, it should be noted that the life-course intervention strategies will not take effect until 30 to 50 years from now, when the current younger population reaches the older stage. However, China has already entered a stage of deep aging. The following measures can be implemented to address pressing current needs: further refining clinical health risk screening services for older populations, and expanding the supply of related professional services such as vision care, oral health care, rehabilitation, and nursing care [61]; facilitating older adults’ easy access to health management education and physical exercise resources; strengthening community- and family-based systems for basic medical services, rehabilitation care, and LTC, in view of the predominant preference among Chinese older adults to live at home; and developing an accessible physical environment conducive to fall prevention, safe mobility, and meeting the daily living needs of older adults. The aim is to reduce the risk of increased frailty and progression to higher levels of dependence, and empower older individuals to maintain greater control over their lives as they enter advanced old age.

### 4.2. The Impact of Longer Disability Duration and Rising LTC Demand

The prediction showed that from 2030 to 2100, the duration of disability among the older population in China will perform a gradual upward trend, accompanied by the growing size of the disabled older population. The condition will undoubtedly impose significant burdens and serious challenges on China, while also forcing the implementation of a series of strategic reforms in relevant fields [62,63].

First, from the perspective of older adults, a longer duration with impairment and dysfunction may imply an accumulation of negative factors and a lack of resources [64]. Examples include increased daily dependence, a sustained rise in health care expenditures, and a deteriorating economic situation [65,66]. Of particular concern is that the low replacement rate of China’s basic pension system will be further highlighted. Although in recent years, China has phased out the special pension scheme for civil servants, established the basic old-age insurance for urban employees, and introduced the basic old-age insurance for urban unemployed residents and rural residents, with monthly average basic pension benefits continuing to rise [67]. These measures are intended to provide financial support to China’s older individuals through a more universal, equitable, and robust system. Yet, nearly 80% of countries globally maintain a pension replacement rate of over 60%. Examples include Japan (62.3%) [68], South Korea (66.4%) [69], and Germany (77%) [70]. In contrast, over the past two decades in China, the country’s pension replacement rate has consistently fallen below the 55% threshold, while exhibiting a downward trajectory [71]. In the absence of improvements to this situation, the prolonged duration of disability will impose heightened financial and caregiving burdens, which in turn will further reduce the effective pension replacement rate for older adults and significantly escalate individual financial strain.

Second, from the perspective of family, it places heavy stress and burden on the family [72]. Family continues to serves as the dominant core field and resource hub of older care services in China. According to data from China’s seventh national population census, the proportion of older individuals living in care institutions nationwide accounts for only 0.73% of the total older population [73]. This is markedly distinct from the older care systems in Western countries, where the proportion is generally above 5% [74,75]. Historically, the social ethics and value logic of state governance shaped by Confucianism’s emphasis on filial piety and familial culture have served as key drivers sustaining this phenomenon [76]. Even as times change and societal conditions evolve, this cultural tradition continues to profoundly influence the care choices of older Chinese adults. However, as Chinese families evolve toward smaller sizes and nuclear tendency, the “4-2-1” inverted pyramid caregiving structure has emerged as a new predominant feature in contemporary society, leading to sustained pressure and gradual erosion of the human and financial resource base underpinning familial support systems. At this point, confronted with the growing number of the older population with impairment and dysfunction and the increasing duration of disability, this network may easily face a severe breakdown crisis. Concurrently, an effective family policy and support system remain underdeveloped, as institutional care has long been the focus of China’s older adult care efforts, while the development of family- and community-based care service supply and payment systems has lagged behind. This will undoubtedly exacerbate families’ caregiving burden and vulnerability.

Finally, from the perspective of society, as a key strategic measure for the country to proactively address population aging and a core mechanism for the socialized diversification of disability risks, China’s long-term care insurance (LTCI) system has fundamental deficiencies. Given the rapid increase in care demand and its projected persistence at high levels in the future, socialized disability risks are expected to become increasingly pronounced Specifically, similar to these countries (e.g., Germany and Japan), China has explored the establishment of LTCI systems to address the expanding care needs of the disabled population. This system’ purposes is to diversify socialized disability risks through a risk-sharing mechanism and avoid societal systemic collapse. The LTCI pilot program was launched in 2016. As of 2024, China LTCI had covered about 180 million people in 49 cities, benefiting 2.6 million people and reducing the burden on the masses by over CNY 80 billion [77]. Nevertheless, the following factors deeply restrict the future feasibility, effectiveness and sustainability of LTCI [78]. In the terms of funding security, a socialized risk-pooling fund has not yet been established, and the system relies heavily on government subsidies, placing primary strain on local fiscal resources [79]; in the terms of service provision, challenges such as homogeneous service offerings, substandard quality, and persistent structural mismatches are often observed. These issues have led to ongoing concerns about the system’s capacity to respond.

In summary, this study contends that mitigating current and future disability risks and alleviating associated care pressures necessitate coordinated action across individual, familial, and societal levels to develop forward-looking and systematic responses. For instance, it is essential to reform and refine the three-pillar old-age insurance system, particularly by strengthening the first pillar and improving basic pension levels for certain older groups to enhance individuals’ financial resilience; advance a family-centered welfare framework and bolster family resilience, through resource infusion and capacity building to better sustain the family’s function in care and related responsibilities; and accelerate the construction of LTCI systems with sound financial mechanisms and high-quality service provision, forming a “systematic buffer” against disability risks to support the sustainable delivery of care in the longevity era. These insights are relevant to LMICs that, similar to China, uphold strong family-oriented traditions but remain inadequately equipped with institutional mechanisms to manage disability risks and care pressure in the longevity era [80].

### 4.3. The Variations in Health and Care Needs Across Age Groups

This research revealed that the risk of disability among older adults accumulates with age at an accelerating rate, particularly among individuals aged 85 and above, leading to a sharp surge in their care needs. Numerous studies have documented this aging pattern [81,82]. For instance, older adults’ capacity to perform activities of daily living declines with age, and this trend is especially prevalent in those aged 80 and above [83,84]. This further provides evidence for the heterogeneity within the older population. The rationale for emphasizing this point is as follows: many countries recently entering the aging stage, often with limited understanding of its complexities, tend to treat older adults as a homogeneous group. China has experienced this process in the past, and certain regions continue to do so today due to imbalanced regional development. As a result, policy recommendations have been overly broad and general, lacking specificity and feasibility, which undermines their implementation effectiveness [85]. In this context, the study argues that focusing on the internal diversity of the older population is crucial not only for China, but also for many LMICs that have recently entered the aging stage or will do so soon.

Thus, this study calls for greater attention to health disparities across different age groups and advocates for the provision of precision-based, categorized, and targeted services for older adults in different age brackets. The goal is to slow the trend of health deterioration and rising disability levels as their age. Specifically, for young-old adults (who have strong and stable functional capacities), services such as health education, appropriate physical activity, and balanced nutrition should be provided, with a focus on maintaining their healthy and vital state as much as possible. For middle-old adults (whose self-care abilities are declining), the focus should shift to minimizing the impact of diseases on individual functions, with targeted interventions including specialized chronic disease management and rehabilitative care to prevent or delay further physical decline. For oldest-old adults (who often experience severe disability and dementia), LTC and palliative care services should be prioritized, with an emphasis on maximizing their quality of life.

### 4.4. The Health Disparities Across Gender Categories

This study showed that Chinese older women have longer lifespans but poorer health conditions in the longevity era. Between 2030 and 2100, both the size and the average duration of the disabled population among this group will be higher than those among older men, and they exhibit a more urgent demand for LTC services of greater quantity and higher intensity. This trend is not unexpected. Several studies have affirmed that men have higher mortality rates and shorter LE than women [86], while women experience higher levels of disability and a greater prevalence of illness [87,88]. Indeed, numerous findings have offered important explanations for the health disparities from various perspectives [89,90]. For instance, these include biological differences between males and females, the overestimation of men’s health status due to survival bias, and the behavior that women are more proactive in health management, which in turn leads to their detection of more health issues. However, these contents do not fully account for the assertion regarding disparities in health conditions and lifespans between men and women [91]. As a larger body of research indicated, it is also one of the key causes of this difference that gender inequalities are driven by social structural factors [92]. Particularly in countries where resource allocation is skewed toward men, gender role norms continue to exert significant influence, and gender-neutral welfare policies remain relatively scarce. These social structural factors and gender inequalities greatly affect women’s adverse health outcomes [93,94]. As Zhang reported, approximately 30% of the gender-based health gap among Chinese residents can be explained by gender inequalities in socioeconomic status [95]. Notably, an in-depth exploration from the perspective of gender inequalities does not mean dismissing the importance of other key factors. Precisely because the factor exists objectively and carries a profound influence, while being intangible, hard to detect, and often overlooked, this study gives greater attention on it.

In China, gender inequalities have a negative impact on the health of older women through the following mechanisms. First, deficient attention is given to women’s health, whether by women themselves, their families, or society as a whole. For instance, women still held conservative attitudes toward their bodies and felt ashamed to seek medical care for gynecological diseases, while stigma surrounding women’s health issues, such as menopause, persisted within families and broader society [96,97]. Second, due to disparities between women and men in employment, education, and economic empowerment, women have access to fewer resources than men do. For example, women exhibit lower wages, pension levels, and coverage rate of basic medical insurance. The pension of older Chinese men was approximately 1.9 times that of older women [98]. There was a significant gender disparity in the coverage rate of China’s Urban Employee Basic Medical Insurance, particularly among those aged 55 and above, with older women having a relatively lower coverage rate [99]. These constrain older women’s capacity to obtain the required health services [100]. Third, it is a demanding task to assume the role of a family caregiver [101]. Common challenges for family caregivers included sleep disturbances, declines in overall health and well-being, heightened feelings of loneliness, and increased depressive symptoms [102,103,104]. In China, this role is predominantly undertaken by women, especially middle-aged and older women. With respect to the proportion of individuals responsible for family care, Chinese women (43.1%) were nearly 24 percentage points higher than men (19.4%); the average caregiver age was 41.2 years, with a higher proportion of older age groups taking primary responsibilities [105]. This role tends to impose greater physical strain and psychological burden on older women. An empirical study indicated that high intensity or continuous caregivers fared poorer health outcomes, and beginning at age 35, caregivers’ health [106].

Therefore, this study holds that it is essential to enhance the public’s scientific understanding of women’s health conditions and eliminate elated stigma; improve access to disease screening and health monitoring services for women, particularly low-income and older women; and recognize the value of informal caregiving as well as their pressure, establishing a family care support system to decline the burden on family caregivers. This will help China lessen some health disparities caused by gender inequalities in the future and also offer insights for many LMICs with cultural and social contexts similar to those of China.

### 4.5. Limitations

To some extent, this study has filled gaps in previous research by providing empirical evidence and policy-relevant insights into the potential longevity risks and care pressures that China may face in the future. Nevertheless, several limitations remain. Firstly, while CLHLS has supported valuable data for health studies on the older population, there is a strong bias towards the oldest-old group in its sample design. This tends to result in an imbalance in the age distribution of the overall sample, which may potentially affect the representative estimation of the disability status among the young- and middle-aged elderly groups. A limitation is that we address oldest-old overrepresentation only by proportional reweighting to the 2020 Census age–sex margins; residual selection within age–sex strata due to survival or attrition may remain because we did not apply longitudinal survival weighting or multistate hazard corrections. Secondly, the classification of disability level in this paper is independently constructed based on the ADL/IADL variable system, which offers greater flexibility and adaptability compared to existing literature. Nevertheless, due to the finite indicators in the scale, the current classification is relatively rough, making it difficult to comprehensively capture the internal heterogeneity of the disability state. Thirdly, although CLHLS data had been publicly released, the update intervals for some survey waves were quite long. The data used in this article, which were published as of 2018, cannot yet reflect the latest trends in population health dynamics.

## 5. Conclusions

Against the backdrop of the longevity era, global awareness of the rising demand for long-term care (LTC) among older adults in low- and middle-income countries (LMICs), as well as the consequent economic and social crises, remains inadequate. These undermine the international community’s synergic efforts to address the systemic risks and challenges hiding in the longevity era and aging societies. To fill this gap, this study takes China, one of the LMICs experiencing a rapid aging process, as research setting, and predicts changes in the health status of older adults and the growth of care demand in China from 2030 to 2100, using a Markov chain health-state transition model informed by the logic of an age-shift algorithm. The goal is to identify the care-related pressure and challenges that China may encounter in the future and to critically examine the insufficiency of existing healthcare and LTC systems. The results indicated that the disabled older population in China will increase to 153 million by 2050, accounting for 43.77% of the total older population; the projected PYD burden shows a sustained rise, with pronounced accumulation among the oldest-old and women. By 2100, adults aged 95 and above are expected to contribute over 20 million PYD, which is eight times the level in 2030, signaling a growing care intensity in extreme old age. Gender disparities persist: in 2100, women aged 85–94 will bear 53.94 million severe-disability PYD, exceeding men by 8.22 million. In conclusion, the pressure on systematic LTC that China may be confronted with in the future will continue to intensify. Thus, there is an urgent need to proactively formulate intervention policies by investing in health promotion throughout the entire life cycle, establishing a multi-level support system encompassing individuals, families, and society, while accounting for age-based internal disparities within the older population and considering the health impacts of gender inequalities. The purpose is to prevent projected demographic trends from evolving into an actual crisis. Additionally, this study also hopes to offer valuable information to a broader range of LMICs to guide the formulation of targeted policies and enhance the delivery of supportive services.

## Figures and Tables

**Table 1:** Total Population and Disabled Older Population in China (10,000 persons, %).

	2030	2040	2050	2060	2070	2080	2090	2100
Total Population-pes	20,727.29	27,674.90	37,166.34	34,532.08	37,610.81	31,158.40	31,486.32	25,422.34
Disabled Older Population-Pes	8238.17	11,332.61	15,281.48	15,059.27	15,956.64	13,907.89	13,549.29	11,510.57
Disabled Older Population-Neu	7949.83	11,015.30	14,960.57	14,848.44	15,844.94	13,859.21	13,501.87	11,470.28
Disabled Older Population-Opt	7661.50	10,697.98	14,639.66	14,637.61	15,621.55	13,615.82	13,264.75	11,268.85
Proportion of Disabled Older Adults (%)-Pes	39.75	40.95	41.12	43.61	42.43	44.64	43.03	45.28
Proportion of Disabled Older Adults (%)-Neu	38.35	39.80	40.25	43.00	42.13	44.48	42.88	45.12

Source: Authors’ calculations based on predictive modeling results.

## Data Availability

All data and analytical results generated in this study are fully incorporated into the article.

## Abbreviations

The following abbreviations are used in this manuscript:

PYD	Person-Years with Disability
LE	Life Expectancy
HLE	Healthy Life Expectancy
LTC	Long-Term Care
LTCI	Long-Term Care Insurance
HICs	High-Income Countries
LMICs	Low- and Middle-Income Countries

## Appendix A

**Table A1:** Projected duration of health states among older adults by age group (years).

Age Group (Years) and Health State								
Total Population	2030	2040	2050	2060	2070	2080	2090	2100
65~74								
Mild Disability	5.4	5.66	5.88	6.1	6.3	6.5	6.71	6.91
Severe Disability	1.19	1.25	1.3	1.35	1.39	1.44	1.48	1.53
75~84								
Mild Disability	4.48	4.69	4.93	5.12	5.33	5.51	5.73	5.93
Severe Disability	1.44	1.5	1.58	1.64	1.71	1.77	1.84	1.9
85~94								
Mild Disability	2.91	3.04	3.17	3.33	3.47	3.61	3.75	3.94
Severe Disability	2.15	2.25	2.35	2.46	2.57	2.67	2.78	2.91
95+								
Mild Disability	1.1	1.14	1.16	1.21	1.26	1.31	1.37	1.43
Severe Disability	2.18	2.25	2.3	2.4	2.49	2.6	2.71	2.84
Male population								
65~74								
Mild Disability	4.41	4.62	4.8	4.97	5.13	5.3	5.47	5.63
Severe Disability	1	1.04	1.09	1.12	1.16	1.2	1.24	1.27
75~84								
Mild Disability	4.23	4.43	4.65	4.84	5.03	5.2	5.41	5.61
Severe Disability	1.15	1.2	1.26	1.31	1.37	1.41	1.47	1.52
85~94								
Mild Disability	2.89	3.02	3.15	3.31	3.45	3.59	3.73	3.92
Severe Disability	1.68	1.76	1.83	1.92	2.01	2.09	2.17	2.28
95+								
Mild Disability	0.93	0.96	0.98	1.02	1.06	1.11	1.15	1.21
Severe Disability	2.1	2.17	2.22	2.31	2.4	2.51	2.61	2.74
Female population								
65~74								
Mild Disability	5.79	6.07	6.3	6.53	6.75	6.97	7.19	7.4
Severe Disability	1.44	1.51	1.57	1.63	1.68	1.74	1.79	1.85
75~84								
Mild Disability	4.26	4.46	4.68	4.86	5.06	5.23	5.44	5.63
Severe Disability	2.59	2.72	2.85	2.96	3.08	3.19	3.31	3.43
85~94								
Mild Disability	3.18	3.33	3.47	3.64	3.8	3.95	4.11	4.31
Severe Disability	2.29	2.4	2.5	2.63	2.74	2.85	2.96	3.11
95+								
Mild Disability	1.23	1.27	1.3	1.35	1.41	1.47	1.53	1.6
Severe Disability	2.13	2.21	2.26	2.35	2.45	2.55	2.66	2.79

Source: Calculated based on projected older population by sex and health state transition probability matrices.
